# Modeling speech imitation and ecological learning of auditory-motor maps

**DOI:** 10.3389/fpsyg.2013.00364

**Published:** 2013-06-27

**Authors:** Claudia Canevari, Leonardo Badino, Alessandro D'Ausilio, Luciano Fadiga, Giorgio Metta

**Affiliations:** ^1^Mirror Neurons and Interaction Lab, Robotics, Brain and Cognitive Sciences Department, Istituto Italiano di TecnologiaGenova, Italy; ^2^Dipartimento Scienze Biomediche e Terapie Avanzate – Section of Human Physiology, University of FerraraFerrara, Italy; ^3^Centre for Robotics and Neural Systems, University of PlymouthPlymouth, UK

**Keywords:** speech imitation, mirror neurons, automatic speech classification, phone classification, acoustic-to-articulatory mapping, speaker normalization, deep neural networks

## Abstract

Classical models of speech consider an antero-posterior distinction between perceptive and productive functions. However, the selective alteration of neural activity in speech motor centers, via transcranial magnetic stimulation, was shown to affect speech discrimination. On the automatic speech recognition (ASR) side, the recognition systems have classically relied solely on acoustic data, achieving rather good performance in optimal listening conditions. The main limitations of current ASR are mainly evident in the realistic use of such systems. These limitations can be partly reduced by using normalization strategies that minimize inter-speaker variability by either explicitly removing speakers' peculiarities or adapting different speakers to a reference model. In this paper we aim at modeling a motor-based imitation learning mechanism in ASR. We tested the utility of a speaker normalization strategy that uses motor representations of speech and compare it with strategies that ignore the motor domain. Specifically, we first trained a regressor through state-of-the-art machine learning techniques to build an auditory-motor mapping, in a sense mimicking a human learner that tries to reproduce utterances produced by other speakers. This auditory-motor mapping maps the speech acoustics of a speaker into the motor plans of a reference speaker. Since, during recognition, only speech acoustics are available, the mapping is necessary to “recover” motor information. Subsequently, in a phone classification task, we tested the system on either one of the speakers that was used during training or a new one. Results show that in both cases the motor-based speaker normalization strategy slightly but significantly outperforms all other strategies where only acoustics is taken into account.

## Introduction

Speech imitation requires the transformation of acoustic information into motor programs to be executed. This task purportedly requires the existence of an auditory-to-motor map (AMM, sometimes referred to as acoustic-to-articulatory map) connecting single instances of both modalities. The most prominent defect caused by a disconnection between auditory and motor maps is that observed in conduction aphasia. Patients with parieto-insular lesions are often characterized by transient language disturbances with relatively fluent spontaneous speech, good comprehension, but poor repetition associated with abundant phonological paraphasias (Bernal and Ardila, 2009). The exact location of the damage that induces this pattern of sensory-motor disconnection was classically associated, by Geschwind, to the arcuate fasciculus. Therefore, conduction aphasia was considered as a physical disconnection between the anterior and the posterior language areas (Catani and ffytche, 2005). However, this idea has been challenged by more recent studies that suggest a cortical origin in the inferior parietal lobule (Fridriksson et al., 2010). Nevertheless, the arcuate fasciculus may still serve in language development by facilitating the repetition of phonological elements in speech, and therefore helping in learning language and monitoring speech (Bernal and Ardila, 2009).

On the other hand, neuroimaging research has defined a dual brain pathways model for speech perception, separating the roles of dorsal and ventral route (Hickok et al., 2011). In this context the dorsal route might be responsible for sensory-motor mapping in speech tasks. Recent studies suggest at least two different brain locations for the sensory-motor interface. One possibility is that the junction between the posterior superior temporal gyrus and the inferior parietal lobule is the seat for the process of sensory-motor conversion (Hickok et al., 2003). Other studies suggest that such an interface might be located in premotor areas instead (Skipper et al., 2007; Iacoboni, 2008). However, absolute brain locations do not matter until we do not define the computational mechanisms involved. In fact, focusing on task-evoked responses in the brain could be misleading. Such an approach suggests a reflexive view of brain functions (Raichle, 2010), ignoring that brain functions involve active information processing for interpreting, responding to and predicting environmental demands (Pulvermüller and Fadiga, 2010; Friston et al., 2011). Active perception encapsulates motor responses and external data encoding in the same functional unit (Fowler, 1986), shifting the focus from task-related activations to processes.

Regarding the sensorimotor conversion process, mirror neurons offer a network-oriented and process-oriented view of how such coordinate transformation may happen. In fact, the mirror system receives the visual representation of actions and transforms them into the motor coding of that same actions (Rizzolatti and Craighero, 2004). The sensory-motor conversion properties of mirror neurons are indeed the result of a tempo-parieto-frontal network of areas (Fogassi and Ferrari, 2010). Furthermore, the mirror neuron theory suggests that visual (or audio) representations of actions that are part of our motor repertoire can exploit an additional inferential process based on the emulation of analogous motor commands in our brain (Grush, 2004). Finally, mirror neurons have been associated to imitation abilities (Iacoboni et al., 1999) but here we only stress their sensory-motor conversion function, which might be a necessary but not sufficient component of imitation behavior.

According to motor theories (Galantucci et al., 2006) as well as sensory theories of speech production (Hickok et al., 2011) a central ontogenetic factor, in building sensory-motor maps, is speech production learning. During early speech learning we generate sounds by controlling our phono-articulatory apparatus. The simple association of a (random) motor command to its sensory (auditory, somatosensory and proprioceptive) effects may explain how sensory-motor maps can be learned (Guenther, 1995, 2006; Kröger et al., 2009). Infants indeed generate and discriminate all possible sounds their articulatory system allows to produce (Werker and Lalonde, 1988; Kuhl, 2004). Interestingly, language-specific abilities start earlier for discrimination (6 months) than for production (10 months). This may suggests that robust input separation is a prerequisite for correct imitation. On the other hand, as soon as language-specific abilities in production are mastered (10 months), the discrimination of foreign sounds soon decline (11 months). This implies a form of pruning of sensory-motor map representations as a function of effective use. These simple facts show that imitation must be present at very early stages of human development and could be the driving factor also in shaping perceptual abilities.

However, by suggesting a developmental and imitative strategy for the acquisition of sensory-motor maps we still have not said much about the mechanisms supporting it. One major problem of cognitive modeling is exactly this. Cognitive models are often vague enough to be always true, and hardly falsifiable. One solution is to build computational models that can be tested on the numerical prediction they imply (Garagnani et al., 2008; Hickok et al., 2011; Hickok, 2012). However, most computational models using a classical neural network approach, although very powerful, use many oversimplifications regarding input coding. In simple terms, these models cannot receive audio streams as input, but rather use a symbolic *ad-hoc* coding. Furthermore, these models are intrinsically characterized by several free parameters for which there is no a priori ground truth. Finally, these models hardly perform human-like classification of speech utterances.

Here, as already proposed in Badino et al. (in press), we aim at a radically different computational approach. Specifically we use state-of-the-art machine learning methods to run functional rather than structural simulations of human behavior. With structural simulation, we intend the modeling of a cognitive process with the best degree of biological substrate plausibility (i.e., simulating inter-areal communication, population activity and even action potential generation). On the other hand, functional simulations start from the assumption that even the most detailed implementation will not satisfactorily simulate real neuronal dynamics and, even when possible, it will lead to an intractably complex new problem. A functional-oriented modeling aims at simulating critical aspect of biology, keeping in mind the need to build reliable and robust systems that could, in the future, substitute human functions in realistic scenarios.

More specifically, we start from neurophysiological research demonstrating how motor knowledge enhances speech classification. Transcranial magnetic stimulation of the motor cortex induces a somatotopical facilitation of the discrimination of speech sounds (D'Ausilio et al., 2009, 2011). Analogous results have been replicated in several labs using different stimulation protocols and tasks (Meister et al., 2007; Möttönen and Watkins, 2009; Sato et al., 2009). Also, the recruitment of the motor system in different speech tasks has been reported with different techniques, methods and material (e.g., Fadiga et al., 2002; Watkins et al., 2003; Binder et al., 2004; Callan et al., 2004; Wilson et al., 2004; Pulvermüller et al., 2006; Shahin et al., 2009; Londei et al., 2010).

**Figure 1:**
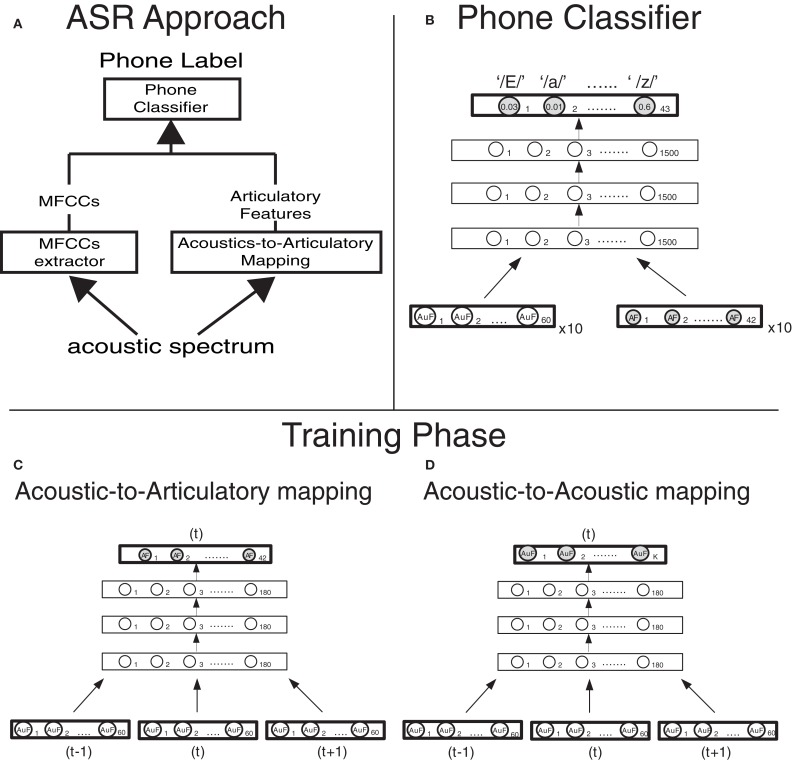
**Computational approach and model design (A) Architecture of the phone classifier**. Ten vectors of 60 Mel-filtered Spectra Coefficients (MFSCs) and, when articulatory features are used (dashed lines), 10 vectors of 42 articulatory features (reconstructed from the MFSCs though the acoustic-to-articulatory mapping, in panel **B**) are fed into a Deep Neural Network. The Deep Neural Network computes the posterior probabilities of each of the 43 phones (output layer) given the acoustic and articulatory evidence and then the most probable phone is selected. **(C)** Implementation details of the deep neural network that carries out the acoustic-to-articulatory mapping for motor-based normalization. **(D)** Implementation details of the deep neural network that carries out the acoustic-to-acoustic mapping for the acoustic normalization.

On the other hand, standard Automatic Speech Recognition (ASR) mostly relies on acoustic data only (Huang et al., 2001). The performance of an ASR system trained and tested on the same voice resembles that of humans if the training data set is sufficiently large and the speech is clean. However, the requirement of a very large speaker-dependent dataset largely limits the usability of speaker-dependent systems. Speaker-independent training datasets are a much preferred option but introduce variations (due, to different speaker gender, accent, speaking style, etc…) that cause the ASR system to learn fragmented acoustic models (rather than few compact models) thus limiting its generalization ability. In critical conditions where the effects of speaker variability combine with those due to environment variability, the ASR performance can be poor where humans excel (see, e.g., Sroka and Braida, 2005). New trends in ASR are considering the use of speech production knowledge in order to increase recognition robustness (see King et al., 2007 for a review). In our previous work (Castellini et al., 2011; Badino et al., 2012a,b; Canevari et al., 2013) we showed that when acoustic features are combined with “reconstructed” articulatory data, classification and speaker-dependent recognition performances improve in noisy and clean speech conditions, respectively. These studies rely on an auditory-motor mapping to recover the articulatory features from the speech acoustics (see Figure 1). Our studies are an example of how the translation of neurophysiological results into machine learning strategies could be an effective approach for the technological advancement of ASR systems.

In the present work we aim at introducing one further learning strategy derived from developmental research on imitation. Our general goal is to obtain a reliable speaker normalization strategy through auditory-motor mapping. To this end, we implemented a speaker normalization procedure via imitation. It is important to point out that the difference between imitation and normalization is alike the difference between “phenomenon” and “mechanism.” In fact, imitation is a pervasive phenomenon that is observed across different domains and complexity levels. On the other hand, normalization is a computational procedure by which imitation might emerge. Normalization is indeed an important aspect for speaker-independent ASR systems. Speaker normalization strategies aim at reducing inter-speaker variability and thus reducing the fragmentation of the acoustic models learned by an ASR system. In general, normalization can be achieved by either explicitly removing some acoustic peculiarities of the speaker (e.g., through Cepstral mean removal, Anastasakos et al., 1994, or vocal tract length normalization, Eide and Gish, 1996), by explicitly mapping the speech acoustics of some speakers into the acoustic domain of a reference speaker (e.g., Huang, 1992), or by creating compact models (i.e., models that are robust across inter-speaker variations) that can then be adapted to the different speakers (this is usually referred to as adaptive training strategy, Anastasakos et al., 1996).

Here we seek to apply normalization by mapping the speech acoustics of different speakers into the motor domain of a reference speaker. In analogy with developmental research, we simulate an infant trying to reproduce utterances produced by other speakers. As it happens in this ecological scenario, the speech of several speakers is mapped onto the motor plans of one listener (the infant). It is important to point out that the present study is not the first that addresses the auditory-motor mapping in a speaker-independent setting (see, e.g., Ghosh and Narayanan, 2011; Hueber et al., 2012). However, in the previous studies a speaker independent auditory-motor mapping is achieved by first learning a speaker-dependent auditory-motor map and then applying acoustic speaker adaptation to make the auditory-motor map speaker-independent, while in the present work the speaker-independent auditory-motor map is directly learnt from multi-speaker data (with speech acoustics of more than two subject and motor data of one single subject).

The utility of our motor-based normalization strategy is tested in a phone classification task by comparing it with purely acoustic normalization strategies (i.e., the speech acoustics of the speakers are mapped into the acoustic domain rather than the motor domain of the listener) and to a no-normalization strategy.

## Materials and methods

### Dataset

The dataset is a subset of the corpus described in (Grimaldi et al., 2008). The corpus consists of simultaneous recordings of Italian speech and electromagnetic articulographic (EMA) signal (plus other types of signals, e.g. ultrasounds that have been ignored in this work) from six Italian speakers all originally from Lecce, Italy. EMA data were recorded with a Carstens AG500 electromagnetic articulograph that tracked the movements of 3 magnetic coils glued on the tongue (tip, blade and dorsum), 1 on each lip, 1 on the upper teeth and 1 on the lower teeth. The sampling rate is set at 200 Hz. Our dataset consists of 3120 words uttered by the five subjects (five females). The five subjects were selected because they uttered the same word type at least three times (the sixth subject data are incomplete). The lexicon of our dataset consists of 72 different word types, either pronounced with a declarative intonation or a question intonation, and 64 pseudoword types. When training and testing the classifier the number of phonemes in the training set ranged between 3332 and 4165, while in the testing set ranged between 833 and 1666 (see section Training and Testing Scenarios for more details on training and testing settings).

### Acoustic feature extraction

Concerning features extraction, for each phone we computed 10 vectors of 20 Mel-filtered spectra coefficients (MFSCs) plus their first and second derivatives (resulting in vectors of 60 MFSCs each). MFSCs were both used as input for the acoustic-to-articulatory mapping (AAM) (which reconstructs articulatory information from speech acoustics) and as acoustic observations for the Deep Neural Network(DNN)-based phone classifier (see below). For phone classification/recognition tasks, Mel-filtered Cepstral Coefficients (MFCCs) are the typically used acoustic coefficients, but it turned out that the DNN-based phone classifier performed best when using MFSCs. This is in agreement with previous work in speech recognition based on DNNs (Mohamed et al., 2012b).

The MFSCs were computed using 20 filter bank channels and a 25 ms Hamming window with a “dynamic shift.” The dynamic shift was due to the fact that phones can have different duration and we wanted the 10 MFSCs vectors to be uniformly distributed over time (in order to have a balanced acoustic description of the phone, see Castellini et al., 2011). First and second derivatives were adjusted to take into account the dynamic shift.

### Motor feature extraction

The x-y trajectories (i.e., the trajectories on the sagittal plane) of the seven coils were first smoothed, using a moving average filter with a 15 ms smoothing window, and then their first and second derivatives were computed for an overall 42 articulatory features (AFs). We imposed the same time window used to compute the MFSCs to average the EMA trajectories, velocities and accelerations in order to have the same sampling rate for both acoustic and articulatory.

### Acoustic-to-articulatory mapping

The use of articulatory information during speech recognition implies the articulatory information to be explicitly or implicitly recovered from the speech acoustics. The explicit recovery is performed through an Acoustic-to-Articulatory Mapping (AAM), typically constructed by learning from simultaneous recordings of speech and articulatory movements.

The AAM was learned by a 4-layer DNN. DNNs are feed-forward neural networks whose parameters are first “pre-trained” using unsupervised training of Deep Belief Networks (Hinton et al., 2006) and subsequently fine-tuned using the back-propagation method. In other words, DNNs are an improved version of Feed-forward Neural Networks that exploits the knowledge of the statistical properties of the input domain [i.e., P(X)] to effectively guide the search for input-output relations [i.e., P(Y|X)]. In general the pretraining phase acts as a strong regularizer in the training of the neural network preventing it from a harmful overfitting (which, indeed, we never observed while training all the DNNs). DNNs have already been successfully applied to the AAM problem (Uria et al., 2011; Badino et al., 2012a,b) and, when combined with Hidden Markov Models, are a state-of-art machine learning strategy for automatic phone recognition (Mohamed et al., 2012a).

Our 4-layer DNN was pre-trained using an “equivalent” 3-hidden-layer Deep Belief Network (Badino et al., 2012a). We first trained the 3-hidden-layer Deep Belief Network in an unsupervised fashion (pre-training phase). Subsequently we transformed its stochastic nodes into deterministic ones and added a layer of linear regressors in order to obtain a DNN. Finally the parameters of the resulting DNN were “fine-tuned” using back-propagation. The DNN net had three consecutive acoustic vectors (60 × 3 MFSCs) as input and outputs a vector of 42 articulatory features, corresponding to the frame on which the acoustic input is centered. All hidden layers have 180 units each (Figure 1B).

An identical DNN was also trained to learn “Acoustic-to-Acoustic” Mappings where the speech acoustics of a speaker were mapped into the speech acoustics of another speaker (see section Normalization Strategies). In that case the input to the DNN consisted of three consecutive acoustic vectors (60 × 3 MFSCs) of one speaker and the output was either the 60 MFSC acoustic vector (of another speaker) or a reduced acoustic vector of MFSCs (ranging from 15 to 27 MFSCs), corresponding to the frame on which the acoustic input is centered. (Figure 1C).

### Phone classification

The phone classifier was a 4-layer DNN. The DNN was pre-trained and trained as the DNN used for AAM, the only difference being that the output activation function is a softmax function (instead of a linear regressor). The input to the DNN consisted of 600 MFSCs (10 frames × 60 MFSCs) plus either the corresponding 420 (10 frames × 42) articulatory features when articulatory features were used or the corresponding reconstructed acoustic features when they were combined with the actual ones (in two out of three acoustic normalization strategies, see section Normalization Strategies). Each hidden layer had 1500 units while the output layer had 43 units, one for each Italian phoneme in the dataset (Figure 1A).

### Training and testing scenarios

In order to test the utility of a motor-based normalization strategy, we trained and tested the different phone classifiers (one for each normalization strategy) in two different scenarios. In the first scenario (henceforth referred to as T1 scenario) the phone classifiers were trained using all the available listener (L) data (consisting of acoustic data, plus motor data when motor normalization was applied) and part of one speaker (S1) acoustic data (either 1/3 or 2/3 of the overall S1 acoustic data), and tested on the remaining S1 acoustic data. Within the T1 setting we varied the amount of S1 data used in the training and testing set. We either used 1/3 of S1 data in the training data (T1_1Tr setting) or 2/3 of S1 data (T1_2Tr setting). In the second scenario (henceforth referred to as T2 scenario) the phone classifiers were trained on the same training data set as in the first scenario (but always using 2/3 of the S1 speaker acoustic data) but tested on data of a speaker (S2) that was not used for training. The testing data of S2 was 1/3 of her overall S2 acoustic data. It is worth to stress that in both scenarios the only articulatory data used (for training only) was that of the listener.

### Normalization strategies

By using the T1 and T2 training and testing settings, where the only articulatory data used (for training only) was that of the listener, we assume that normalization (by imitation) is used by the listener (i) when she learns to discriminate phones and (ii) when she either discriminates new speech from a known speaker (i.e., already present in the training set, T1 scenario) or speech of an “unknown” speaker, i.e., who did not contribute to the listener phone discrimination learning (T2 scenario). Normalization always consists in mapping the speaker acoustic data to either the acoustic or articulatory data of the listener (the reference subject). Concerning the motor data, only the actual articulatory data of the listener is available. For each listener we considered all the possible combinations of subjects involving that listener (4 pairs in the T1 setting and 12 triplets in the T2 setting) and then averaged the results.

We experimented with 5 different normalization strategies: 1 no-normalization strategy, 1 motor normalization, and 3 different kinds of acoustic normalization. They were both trained and tested in the T1 and T2 settings (see Table 1) resulting in 10 different classifiers (each of them named concatenating the name of the normalization strategy with that of the training and testing setting). The following is a detailed description of them:

- *NoNorm*. No normalization and no articulatory information are used. The listener discriminates new phones produced by the speaker (i.e., new instances of a phoneme that were not heard by the listener) on the basis of her knowledge about the acoustic correlates of the phonemes learnt from both the listener and the S1 speaker speech.
- *MotorNorm*. With this normalization we mimic the case where the listener normalizes the speaker acoustics by learning to recover her own AFs from the speaker acoustics. We first learned the AAM to map the acoustic features of both the listener and the speaker into the articulatory space of the listener. The types of input-output pairs used to train the DNN performing AAM are shown in Table 3. When feature vectors of two different subjects had to be paired, i.e., when the input features where the S1 speaker MFSCs and the output features were the corresponding listener AFs, the speaker acoustic features and the listener AFs were extracted from the same phoneme of the same word type. The feature sets used by the phone classifiers during training and testing are shown in Table 2.

**Table 1:**
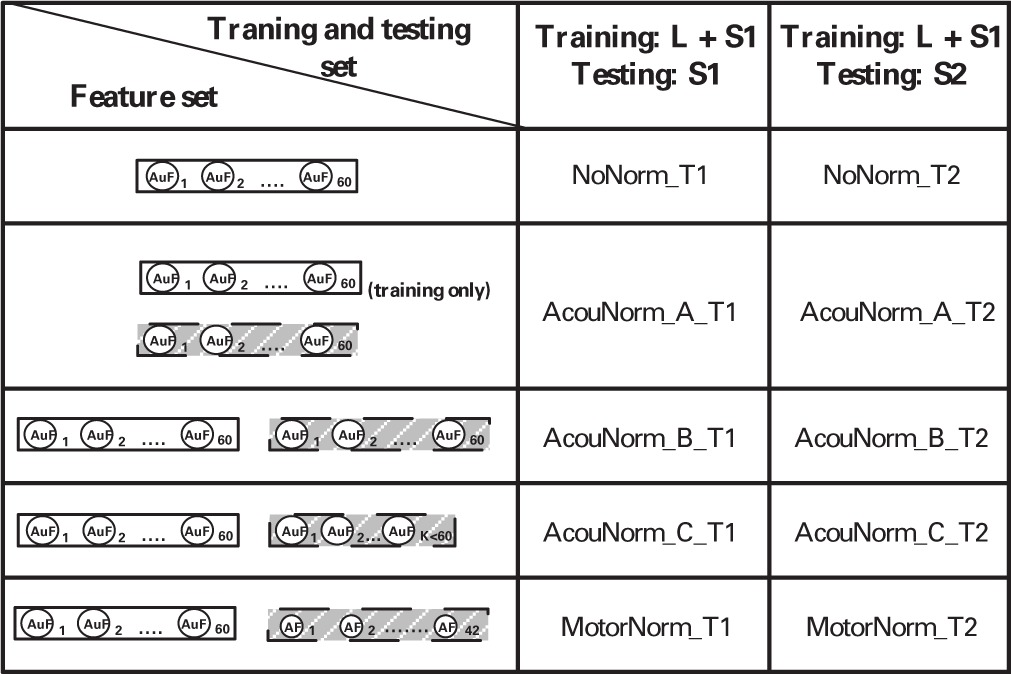
**Normalization strategies and training and testing settings**.

*The combination of five normalization strategies and two main training and testing scenarios results in 10 different phone classifiers. Acoustic features (AuFs) in bold-line rectangular are the actual 60 MFSCs. Acoustic features in shadowed dashed-line rectangles are the reconstructed audio features in the listener domain. They can be all 60 MFSCs as in AcouNorm_A and AcouNorm_B or the K first MFSCs as in AcouNorm_C. Articulatory features (AFs) in shadowed dashed-line rectangles are the 42 reconstructed listener articulatory features. In the NoNorm scenario no normalization is applied while in AcouNorm_A the listener acoustic features reconstructed from speaker S1 are used together with the actual ones only during training*.

**Table 2:** **Feature set of all the phone classifiers**.

**Scenario ID**	**Training set**	**Testing set**
NoNorm_T1	*Audio_L_* + *Audio*_*S*1_	*Audio*_*S*1_
NoNorm_T2	*Audio_L_* + *Audio*_*S*1_	*Audio*_*S*2_
AcouNorm_A_T1	$Audio_{L}+\overset{\leftarrow }{Audio_{S1}}$	$\overset{\leftarrow }{Audio_{S1}}$
AcouNorm_A_T2	$Audio_{L}+\overset{\leftarrow }{Audio_{S1}}$	$\overset{\leftarrow }{Audio_{S2}}$
AcouNorm_B_T1	$Audio_{L}\bar{Audio_{L}}+Audio_{S1}\overset{\leftarrow }{Audio_{S1}}$	$Audio_{S1}\overset{\leftarrow }{Audio_{S1}}$
AcouNorm_B_T2	$Audio_{L}\bar{Audio_{L}}+Audio_{S1}\overset{\leftarrow }{Audio_{S1}}$	$Audio_{S2}\overset{\leftarrow }{Audio_{S2}}$
AcouNorm_C_T1	$Audio_{L}\bar{redAudio_{L}}+Audio_{S1}\overset{\leftarrow }{redAudio_{S1}}$	$Audio_{S1}\overset{\leftarrow }{redAudio_{S1}}$
AcouNorm_C_T2	$Audio_{L}\bar{redAudio_{L}}+Audio_{S1}\overset{\leftarrow }{redAudio_{S1}}$	$Audio_{S2}\overset{\leftarrow }{redAudio_{S2}}$
MotorNorm_T1	$Audio_{L}Motor_{L}+Audio_{S1}\bar{Motor_{LfromS1}}$	$Audio_{S1}\bar{Motor_{LfromS1}}$
MotorNorm_T2	$Audio_{L}Motor_{L}+Audio_{S1}\bar{Motor_{LfromS1}}$	$Audio_{S2}\bar{Motor_{LfromS2}}$

*L is the listener and is the only subject whose actual motor data (used for Acoustic-to-Articulatory Mapping) is available. S1 is the speaker whose data is always used during training and is tested in the T1 training and testing setting. S2 can only be tested (T2 setting). Audio_L_, Audio_S1_, and Audio_S2_ are the vectors of actual acoustic features (actual MFSCs) of the listener, speaker S1 and speaker S2 respectively. $\overset{\leftarrow }{Audio_{S1}}$ and $\overset{\leftarrow }{Audio_{S2}}$ are the MFSC vectors of speaker S1 and S2 respectively mapped into the listener acoustic domain (using the acoustic-to-acoustic mapping). $\overset{\leftarrow }{redAudio_{S1}}$ and $\overset{\leftarrow }{redAudio_{S2}}$ are the MFSC vectors of speaker S1 and S2 respectively mapped into the listener reduced acoustic domain. $\bar{Audio_{L}}$ are the MFSC vectors of the listener mapped onto her own acoustic domain. $\bar{redAudio_{L}}$ refer to the reduced version of $\bar{Audio_{L}}$. $\bar{Motor_{LfromS1}}$ and $\bar{Motor_{LfromS2}}$ are the AFs of the listener reconstructed from S1 and S2 respectively using the Acoustic-to-Articulatory mapping. No testing acoustic data were used in the training set, even when the speaker in the testing set was also in the training set (e.g., NoNorm_T1)*.

Note that the phone classifier is trained using reconstructed AFs rather than actual AFs (especially when paired with the listener audio) because the testing is performed on recovered AFs (paired with acoustic features). Since the reconstruction of AFs is far from being perfect (partly because of the limited data available for AAM learning), the use of actual AFs for training and of recovered AFs for testing, implies the use of different data types with different probability distributions which would violate the working assumption of almost all supervised machine learning strategies and usually results in poor performance.

- *AcouNorm_A*. We carried out a normalization at the acoustic level (an acoustic feature normalization of the type proposed in, e.g., Huang, 1992). The speaker acoustic features were mapped into the corresponding (i.e., belonging to the same phoneme of the same word type) listener acoustic features. For analogy with the motor normalization case we will referred to the mapped speech acoustic as “reconstructed” (listener) acoustic features. To learn the “acoustic-to-acoustic mapping” we trained a DNN using the S1 speaker acoustic features as input and the corresponding listener acoustic features as output (Table 3). The phone classifier was trained using the listener's actual acoustic features and the listener acoustic features reconstructed from S1 acoustics. It was tested using the listener acoustic features reconstructed from either the remaining S1 audio or S2 audio (depending on the training and testing setting) (Table 2).
- *AcouNorm_B*. In this strategy we normalized the speaker speech acoustics (i.e., reconstructed the listener acoustics from the speaker acoustics) as in AcouNorm_A, but in this case the reconstructed acoustic features were paired with the actual ones (e.g., the actual acoustic features of S1 were paired with the listener acoustic features recovered from S1 acoustics, see Table 2). Since this strategy requires <listener actual acoustic features, listener reconstructed acoustic features > pairs, the acoustic-to-acoustic mapping must be different from that of AcouNorm_A. To learn the acoustic-to-acoustic mapping we trained a DNN using as input the acoustic features of either listener or S1 (and not just of S1 as in AcouNorm_A) and as output their corresponding listener acoustic features (Table 3). Thus, in all examples where the input features were the listener acoustic features, the input and the output features were exactly the same and for those examples the DNN had to approximate an identity function. The feature sets used by the phone classifier are described in Table 2.
- *AcouNorm_C*. This normalization strategy is identical to AcouNorm_B with the only difference being a reduced set of reconstructed acoustic features (see Tables 2, 3). The acoustic normalization was carried out on the smallest set of acoustic features that contained an equal or slightly larger amount of discriminative information than the actual AF set. As for AcouNorm_B, the input features of the phone classifier consisted of reconstructed acoustic features paired with the corresponding actual acoustic features. In order to search for an acoustic feature set that had an amount of discriminative information comparable to that of the set of actual AFs we first computed for each listener the classification error of the phone classifier that only used actual AFs. The classifier was trained either on 1/3 or 2/3 of the listener actual articulatory data and tested on the remaining listener actual articulatory data. Then we selected the shortest vector of MFSCs that produced a classification error equal or smaller than that produced by the actual articulatory features. This second phone classifier was trained on either 1/3 or 2/3 of the listener acoustic data (with reduced feature set) and tested on the remaining acoustic data (with the same reduced feature set). The acoustic feature sets were reduced by keeping the first (out of 20) mel-filtered spectra coefficients (plus their first and second derivatives) and discarding all the other coefficients. The number of reconstructed acoustic features turned out to range from 15 to 27 features (while the full features set counts 60 features) and produced a 46.8% overall classification error, while the full actual AF set produced a 51.3% overall classification error. Finally we carried out the acoustic normalization using the reduced acoustic features. The speaker and listener acoustic features were mapped onto the corresponding reduced listener acoustics (Table 3). The phone classifier was trained and tested using pairs of actual and recovered acoustic vectors as in AcouNormB, with the only difference that in this case the set of recovered acoustic feature was reduced (Table 2).

**Table 3:** **Feature sets for training and testing of the acoustic-to-articulatory and the acoustic-to-acoustic mappings**.

**Feature sets**			
**Normalization**	**Training**	**Testing in T1**	**Testing in T2**
AcouNorm_A	*Audio*_*S*1_ - *Audio_L_*	$Audio_{S1}-\overset{\leftarrow }{Audio_{S1}}$	$Audio_{S2}-\overset{\leftarrow }{Audio_{S2}}$
AcouNorm_B	*Audio_L_* - *Audio_L_ Audio*_*S*1_ - *Audio_L_*	$Audio_{S1}-\overset{\leftarrow }{Audio_{S1}}$	$Audio_{S2}-\overset{\leftarrow }{Audio_{S2}}$
AcouNorm_C	*Audio_L_* - *redAudio_L_ Audio*_*S*1_ - *redAudio_L_*	$Audio_{S1}-\overset{\leftarrow }{redAudio_{S1}}$	$Audio_{S2}-\overset{\leftarrow }{redAudio_{S2}}$
MotorNorm	*Audio_L_* - *Motor_L_ Audio*_*S*1_ - *Motor_L_*	$Audio_{S1}-\bar{Motor_{LfromS1}}$	$Audio_{S2}-\bar{Motor_{LfromS2}}$

*This table shows the input-output feature pairs for training and testing the DNNs performing acoustic-to-articulatory mappings and acoustic-to-acoustic mapping*.

We adopted three different but complementary acoustic normalization strategies as each of them can provide useful information in the comparison with motor normalization. The AcouNorm_A normalization follows the typical approach of the normalization/adaptation strategies used in ASR where the normalized/adapted features (or the adapted statistical models, in model adaptation techniques) are used as the only feature set and are not paired with the actual, i.e., not normalized, features (as it was the case for AcouNorm_B and AcouNorm_C). However, if the acoustic-to-acoustic mapping is not sufficiently accurate (e.g., because the dataset used to learn it is relatively small) there is a loss of (discriminative) information content in the transformation from actual to normalized acoustic features. In that case the comparison between AcouNorm_A and MotorNorm would have a strong bias in favor of MotorNorm where a not reduced acoustic information content is guaranteed by the actual acoustic features (which are paired with the reconstructed AFs).

To avoid that potential bias we can simply pair the normalized acoustic features with the actual ones, as we did in AcouNorm_B. However, an AcouNorm_B vs. MotorNorm comparison does not take into account that the reconstructed AFs set cannot contain all the information contained by the “reconstructed” acoustic features. In fact, not only the AFs reconstruction can be “lossy” as the acoustic reconstruction, but the actual AFs (or perfectly reconstructed AFs) have much less discriminative information content than the actual acoustic features. For example, there is no information about the consonant manner of articulation (e.g., nasalization) in the AFs we used. This is mainly due to technical difficulties in recording all the relevant articulatory features of the vocal tract.

Under this perspective the AcouNorm_C vs. MotorNorm comparison is the most unbiased in that the discriminative information content of the acoustic and motor features used for normalization is comparable. For that reason we will mainly focus on the AcouNorm_C vs. MotorNorm comparison when comparing the results of the acoustic and motor normalizations.

Finally it is important to point out that the motor normalization we carried out relies on an acoustic bootstrap which is a kind of normalization. When creating the training dataset for the Acoustic-to-Articulatory Mapping we paired vectors of S1's MFSCs with vectors of listener's AFs that belonged to the same phoneme of the same word type. That implies that during training the listener aligns segments of the speaker speech with segments of her vocal tract movements that produced the same phone in the same context. Such alignment can only act in the acoustic domain.

## Results

Table 4 shows the phone classification error rates averaged over all listener cases of the 10 phone classifiers (5 normalization strategies × 2 training and testing settings). The MotorNorm outperforms all other strategies, except AcouNorm_B in the T2 and T1_1Tr settings. The error reduction produced by MotorNorm is always significant (*p* = 0.05) according to the McNemar's test (McNemar, 1947; Gillick and Cox, 1989) pooling together all listener cases, with the exception of Motor Norm vs. AcouNorm_B in the T2 and T1_1Tr settings. In those cases MotorNorm becomes significantly better when the speaker 2 case is removed.

**Table 4:** **Overall phone classification error rate**.

	**T1_1Tr%**	**T1_2Tr%**	**T2%**
**NoNorm**	25.2 (25.3)	20.9 (21)	26.6 (27.1)
**AcouNorm_A**	29.9 (29.8)	26.7 (27.2)	34.3 (34.4)
**AcouNorm_B**	23.8 (24.1)	19.8 (19.9)	25.9 (26.6)
**AcouNorm_C**	24.6 (24.7)	20.4 (20.5)	26.5 (26.9)
**MotorNorm**	23.9 (23.6)	19.7 (19.4)	26.1 (26.2)

*Phone classification error rates averaged over all listener cases of all normalization strategies in all training and testing settings. In parenthesis the phone error rate values obtained by removing listener 2*.

**Figure 2:**
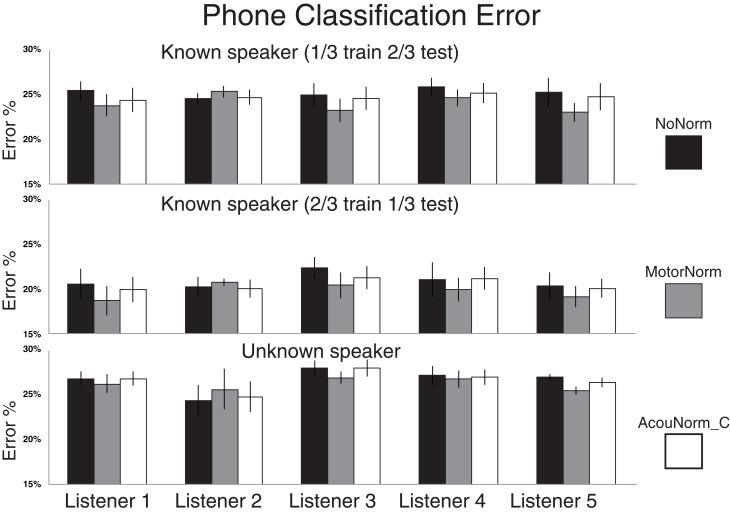
**Phone classification error rates for NoNorm, MotorNorm, and AcouNorm_C. Top panel:** Phone classification error rate in the T1_1Tr setting (all listener data plus 1/3 of the S1 speaker data used for training, and 2/3 for testing). The classification error rate of each listener is averaged over the four listener-speaker pairs. **Middle panel**: Phone classification error rate in the T1_2Tr setting (all listener data plus 2/3 of the S1 speaker data used for training, and 1/3 for testing). The classification error rate of each listener is averaged over the four listener-speaker pairs. **Bottom panel**: Phone classification error rate in the T2 scenario(all listener data plus 2/3 of the S1 speaker acoustic data used for training, and 1/3 of S2 speaker data used for testing) The classification error rate of each listener is averaged over all 12 < L listener, S1 speaker, S2 speaker > triplets.

**Figure 3:**
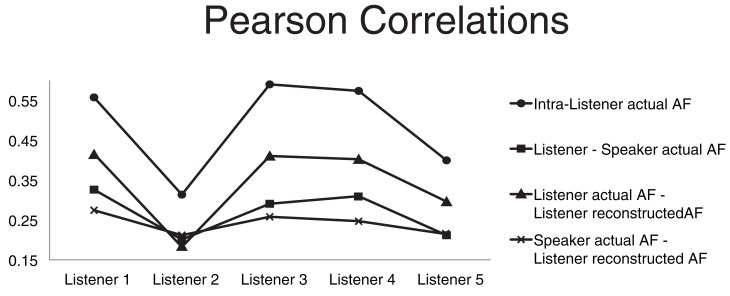
**Four average correlations**. The four Pearson product moment correlation coefficients are computed for each listener and averaged over all speakers. Average correlation between articulatory features (AFs) of the same subject extracted from different instances of the same word type (circles). Average correlation between the listener actual AFs and the corresponding speaker actual AFs (squares). Average correlation between the listener actual AFs and corresponding AFs recovered through acoustic-to-articulatory mapping (triangles). Average correlation between the speaker actual AFs and the corresponding AFs recovered from the same speaker acoustics (crosses).

The relative phone error reduction produced by the motor normalization with respect to the no-normalization strategy was 4.9% in the T1_1Tr setting, 5.4% in the T1_2Tr setting and 1.8% in the T2 setting. Table 4 also shows the results when the listener 2 case was removed. Removing listener 2 was motivated by the fact that the success of the motor normalization clearly depends on the accuracy in the reconstruction of the listener AFs. If such accuracy is below a certain threshold then the reconstructed AFs have no utility or can even been harmful. This is certainly the case of listener 2 whose AF reconstruction is far less accurate than that of the other listeners (see below, and Figures 2, 3). When removing listener 2, the relative error reduction produced by MotorNorm over NoNorm was 6.7% (T1_1Tr), 7.2% (T1_2Tr) and 3.4% (T2).

MotorNorm and the strongest acoustic normalization, AcouNorm_B, showed comparable results, with MotorNorm slightly outperforming AcouNorm_B in T1_2Tr and in both T1 and T2 settings when the listener 2 case was removed.

AcouNorm_A turned to be by far the worst strategy (even worse than the baseline NoNorm). In the most “fair” comparison between motor normalization and acoustic normalization, i.e., the MotorNorm vs. AcouNorm_C comparison, the relative phone error reduction produced by MotorNorm was 2.9% (T1_1Tr), 3.5% (T1_2Tr) and 1.5% (T2). When listener 2 was removed, it raised to 4.2% (T1_1Tr), 5.1% (T1_2Tr), 2.6% (T2). Figure 2 shows the phone error rates of NoNorm, MotorNorm and AcouNorm_C for each listener and in all training and testing scenarios. MotorNorm significantly outperforms (according to the McNemar's test, *p* = 0.05) the NoNorm and the AcouNorm_C strategies in each listener case, with the exception of the listener 2 case, in all three training and testing.

As expected, a larger amount of the S1 speaker acoustic data in the training set produces a lower error rate for all strategies cases as it can be observed in Table 1 and Figure 2. The increase in error reduction produced by motor-based normalization with respect to the acoustic baseline NoNorm (absolute increase: +0.5%) can be due to the fact that the AF reconstruction is more accurate because more training data were available to learn the AAM. Thus, the amount of data used to learn the AAM can affect the impact of the motor normalization. This also applies to all other acoustic normalization strategies.

With the goal of discovering possible relations between the impact of motor-based normalization and intra- and inter-subject properties/relations in the motor domain we compared four different Pearson product-moment correlations (Figure 3). The first correlation (circle markers) is the correlation between AFs of the same subject extracted from different instances of the same word type. It can be seen as a measure of the coherence of the motor behavior of the subject. The average correlation between the listener actual AFs and the speaker corresponding AFs (square markers) is a measure of the motor similarity between a listener and all the other subjects. The correlation between the listener actual AFs and corresponding recovered AFs from the speaker acoustics (triangle markers) is a measure of how accurately the listener is able to mimic actions when listening to someone else's speech (it is also the measure usually applied to evaluate the accuracy of the motor reconstruction). The last correlation between the speaker actual AFs and the corresponding AFs recovered from the same speaker acoustics (cross markers) is a measure of the ability of the listener in recovering the speaker motor gestures given the speaker acoustics.

The graph of the first correlation shows that subject 2 has a much lower coherence than all other subjects, which can partly due to well-known technical problems in electromagnetic articulograph recordings (Richmond et al., 2011). Subject 2 is also the subject whose AF reconstruction has the lowest accuracy (which we hypothesize can be due to the “lack of coherence” in the articulatory data of subject 2).

Comparison of the third and fourth correlation shows that the reconstructed AFs are more correlated to the listener actual AFs than the speaker actual AFs. The comparison simply confirms that the motor-based normalization strategy imposes a reconstruction bias toward the listener AFs rather than the speaker AFs.

The second correlation measures the motor similarities between the listener and the speakers. We used that correlation to investigate whether the relative classification accuracy increase, produced by the motor-based normalization strategy (with respect to the baseline) in the T1 scenarios, was correlated to motor similarities between listener and speaker. We did not find any significant correlation.

## Discussion

The experimental results presented in the previous section show the phone classification accuracy increase due to the use of motor information for speaker normalization over the case where no normalization is applied (the motor-based normalization strategy produced up to a 7.2% relative classification error reduction). We expect that such improvement would be more dramatic if we had articulatory features that fully describe the vocal tract behavior. A full description of the behavior of the vocal tract is a technological challenge and so far we rely on (noisy) articulatory features that miss relevant information such as the consonant manners of articulation. An idea of the amount of discriminative information lost by the articulatory features we used is given by the classification accuracy of a phone classifier only trained on actual articulatory features. Its average phone classification error rate was 51.3% (when using 2/3 of the listener articulatory data for training), which is much higher than that of the same phone classifier trained on acoustic features only (which turned out to be 24.4%).

This poor description of the vocal tract behavior also affects the comparison between motor normalization and acoustic normalization. When performing the Acoustic-to-Articulatory mapping we a-priori know that critical information will be lost, while that does not apply to the “acoustic-to-acoustic” mapping of an acoustic feature normalization. Despite this strong bias the motor normalization showed a comparable performance with respect to its acoustic counterpart (AcouNorm_B), and actually a small but significant improvement when we removed a case (listener 2 case) where the reconstruction of the articulatory features was very poor (most probably due to technical problems occurred during the recording of the articulatory movements of the subject).

The strong bias in favor of the acoustic normalization can be removed by reducing the set of acoustic features used for normalization to a set that encodes an amount of discriminative information comparable to that of the articulatory feature set (as we did for the AcouNorm_C strategy). Once that bias was removed the supremacy of our motor normalization over its acoustic counterpart was more evident, consistent and statistically significant.

The accuracy increases produced by the motor normalization strategy with respect to both a no-normalization strategy and a “corresponding” acoustic normalization are not outstanding (the largest relative error reduction is 7.2%, while the absolute error reduction is slightly larger than 1%) but their consistency over subjects and their statistically significance support our neurophysiological research suggesting a possible role of the motor system in speech classification tasks.

From a technological perspective the results of this paper can be seen as an incentive to explore new and more powerful normalization techniques that exploit the articulatory domain (possibly better than the normalization strategy we proposed). It is worth to point out that there exist other types of acoustic normalization (e.g., vocal tract length normalization) different from those we experimented. These alternative normalizations could eventually be more successful than the motor normalization we proposed but they would not guarantee a fair comparison between a motor-based and an acoustic-based strategy. Indeed the type of acoustic normalization we applied is the exact acoustic counterpart of the motor-based normalization strategy we proposed. In one case we map the acoustic space of the speaker onto the acoustic space of the listener, while in the other case we map it onto her motor space.

From a theoretical standpoint, this is a critical test, we demonstrate that speaker normalization seems to better rely on a motor rather than an acoustic normalization. However, we must not forget that a speaker-independent motor normalization (i.e., a normalization that allows a listener to recover her own vocal tract motor plans from someone else speech acoustics) is not purely motoric, since it can only be learnt if an acoustic bootstrap is carried out first. That acoustic bootstrap is a kind of normalization that allows to link the speech sounds (e.g., phones) of a speaker to the articulatory movements of the listener that would produce speech sounds belonging to the same phonological categories.

Such acoustic bootstrap is not the only requirement needed to carry out motor normalization. The success of a motor-based normalization, and more in general, the successful use of measured articulatory features, strongly depends on our ability to accurately reconstruct them from the speech acoustics of the speaker. A poor reconstruction of the articulatory features cannot only make the (reconstructed) articulatory features useless but even harmful (as it happened in our experiments with the listener 2 case).

The motor normalization we proposed can be seen as the result of an imitation process where the listener builds a speaker-independent auditory-motor map by imitating the other's speech. This idea is derived from current theories of sensory-motor map acquisition during development, as discussed in the introduction. In this sense, speaker normalization could be seen as the ability to learn a common motor-based template, which fits most of the speech input we encounter in life. Such a template, in our working hypothesis, can be extracted via imitation. In fact, children can continuously adapt motor production to align the resulting auditory effects to the acoustics of a reference model. They implicitly project auditory distances and differences onto their motor space. In this sense, other people's voice can be readily converted into a common motor template.

In all our experiments we considered ecological scenarios where it was assumed that only the speech acoustics of the speaker was available during recognition. Motor information could only be reconstructed from acoustics through an acoustics-to-articulatory mapping. From a computational perspective one may wonder why reconstructed articulatory information improves phone classification accuracy. The reconstructed articulatory features do not provide new information but are the result of a transformation of the acoustic domain carried out by the acoustic-to-articulatory mapping. Such transformation ties the surface level of speech, i.e., the speech acoustics, to its hidden causes, i.e., the speech production processes, which are commonly held to compactly encode all the phonetic differences (see King et al., 2007; Badino et al., in press).

It cannot be excluded that alternative transformations of the acoustic space that do not rely on any knowledge of the speech production process may be equally successful. However, from a developmental perspective it would not be clear why the infant learner should not exploit a powerful tool like the auditory-motor map that naturally builds up during development. The developmental stages involved in the speech competence (outlined in the introduction) clearly speak for a recursive interaction between speech production and perception.

The experiments we presented in this paper were carried out in clean speech conditions. However, there is experimental evidence showing that the role of motor information becomes more essential in critical conditions (e.g., when speech is noisy; Castellini et al., 2011; D'Ausilio et al., 2011; Mitra et al., 2012) or not clearly articulated (as in dysarthria, Rudzicz, 2011). Future corpora with simultaneous recordings of audio and articulatory movements in diverse speaking styles (e.g., spontaneous conversational speech, Lombard speech) will need to take this fact into account. In fact, the introduction of larger variability in the data sets seems a necessary requirement to investigate the full potential of articulatory information for speech recognition (and perception). The few and small corpora available at present do not even allow to fully understand the impact of articulatory information in a speaker-independent scenario where hundreds of speakers are involved.

The present work seems in line with the utility of motor information in multi-speaker scenarios. However, the exact relation between the impact of motor information and the different kinds of variability would require corpora containing a much larger variability (in terms of gender, accent, etc.). Unfortunately, recording the articulatory movements of a speaker is much more time consuming than simply recording her audio, thus the creation of new corpora of articulatory data with hundreds of speakers does not seem easily attainable. A viable alternative solution would consist in recording tens of selected “representative” speakers that would cover as much inter-speaker variability as possible. Current neurophysiological research in our lab aims at defining the speaker characteristics that maximize the efficacy of motor activations in speech classification tasks. These results will hopefully translate in further useful principles to export in ASR research. Namely, we believe that maximizing the efficacy of motor knowledge is the key area of research for future robust speaker independent ASR Systems.

### Conflict of interest statement

The authors declare that the research was conducted in the absence of any commercial or financial relationships that could be construed as a potential conflict of interest.
